# Primary Mid-ureter Squamous Cell Carcinoma: A Diagnostic Conundrum in the Absence of Traditional Risk Factors

**DOI:** 10.7759/cureus.96180

**Published:** 2025-11-05

**Authors:** Saad Masood, Sanya Caratella, Asrit Babu

**Affiliations:** 1 Urology, York and Scarborough Teaching Hospitals NHS Foundation Trust, York, GBR

**Keywords:** mid-ureter cancer, risk factor, scc of ureter, smoker, squamous cell carcinoma, squamous cell neoplasm, tcc, upper urinary tcc, ureteric cancer, urologic cancer

## Abstract

Primary squamous cell carcinoma (SCC) of the ureter is an exceedingly rare entity, accounting for only 1-1.6% of upper urinary tract tumors. It is typically associated with chronic irritation, infection, or urolithiasis and often presents at an advanced stage with a dismal prognosis. We report the case of an 81-year-old male presenting with right flank pain and radiological evidence of hydronephrosis. Imaging identified a mid-ureteric mass without distant metastasis. The patient underwent robot-assisted laparoscopic nephro-ureterectomy with pelvic lymph node dissection, achieving negative surgical margins. Histopathology confirmed moderately differentiated SCC staged as pT3N0R0V0. This case highlights the diagnostic challenge of ureteric SCC in the absence of traditional risk factors and suggests that surgical resection alone may achieve favorable short-term outcomes in select patients.

## Introduction

Upper urinary tract carcinomas represent a small but clinically important subset of renal malignancies, accounting for roughly 5-7% of kidney tumors and 5-10% of all urothelial cancers [1], with an estimated annual incidence of about 1-2 cases per 100,000 in Western population [2].

Most tumors of the upper urinary tract are urothelial in origin; pure squamous cell carcinoma (SCC) of the ureter is exceedingly rare, reported to constitute approximately 1-1.6% of upper tract urothelial neoplasms. Because SCCs are often high-grade and present at an advanced stage, they are associated with a poor prognosis compared with conventional urothelial carcinoma [3].

Several longstanding clinical associations and putative etiological factors have been described for SCC of the renal pelvis and ureter. Chronic urothelial irritation-most commonly from long-standing urolithiasis, chronic infection, and hydronephrosis frequently implicated in squamous metaplasia and subsequent malignant transformation [4]. A history of analgesic abuse, prior pelvic irradiation, and chronic inflammatory states have also been reported in older series and case reports. Some anatomical anomalies such as horseshoe kidney appear to have a higher reported incidence of squamous or variant upper tract malignancies in case reports and small series, likely related to stasis, recurrent infection, and stone disease in these kidney [5].

From an imaging perspective, computed tomography urography (CTU) is now the principal cross-sectional investigation for suspected upper tract malignancy because of its high sensitivity for detecting filling defects, mural thickening, and evidence of extra-ureteral extension (including features such as complete ureteric obstruction). CTU findings importantly guide staging and operative planning, and can detect occult ureteral obstruction that is otherwise clinically silent [6].

We report an 81-year-old male presenting with flank pain, where robotic nephro-ureterectomy achieved clear margins despite advanced local staging (pT3). This case highlights the role of CT urography in detecting occult ureteral obstruction and underscores the need for vigilance in evaluating unexplained ureteric obstruction and re-examining therapeutic paradigms in this aggressive malignancy.

This article was previously presented as a meeting abstract at the Global conference of surgery and anesthesia, 25-27 September 2025.

## Case presentation

An 81-year-old gentleman presented with right flank pain for a few days. He did not have any lower urinary tract symptoms or fever. He denied any nausea, weight loss, or any other constitutional symptoms. Comorbidities included ex-smoker, previous myocardial infarction, stroke, and benign prostatic hyperplasia (BPH).

The blood profile indicates mild anemia (low Hb), slightly elevated creatinine and urea, suggesting reduced kidney function, borderline high potassium, and an estimated glomerular filtration rate (eGFR) of 47 (slightly decreased from baseline 51), consistent with early-stage renal impairment (Table 1).

**Table 1:** Haematological results Hb: hemoglobin; eGFR: estimated glomerular filtration rate.

Parameter	Result	Reference Range
Hb	125 g/L	130–180 g/L
Creatinine	123 µmol/L	59–104 µmol/L
Urea	9.4 mmol/L	2.5–7.8 mmol/L
Potassium	5.6 mmol/L	3.5–5.3 mmol/L
eGFR	47 (Baseline: 51)	—

**Figure 1:**
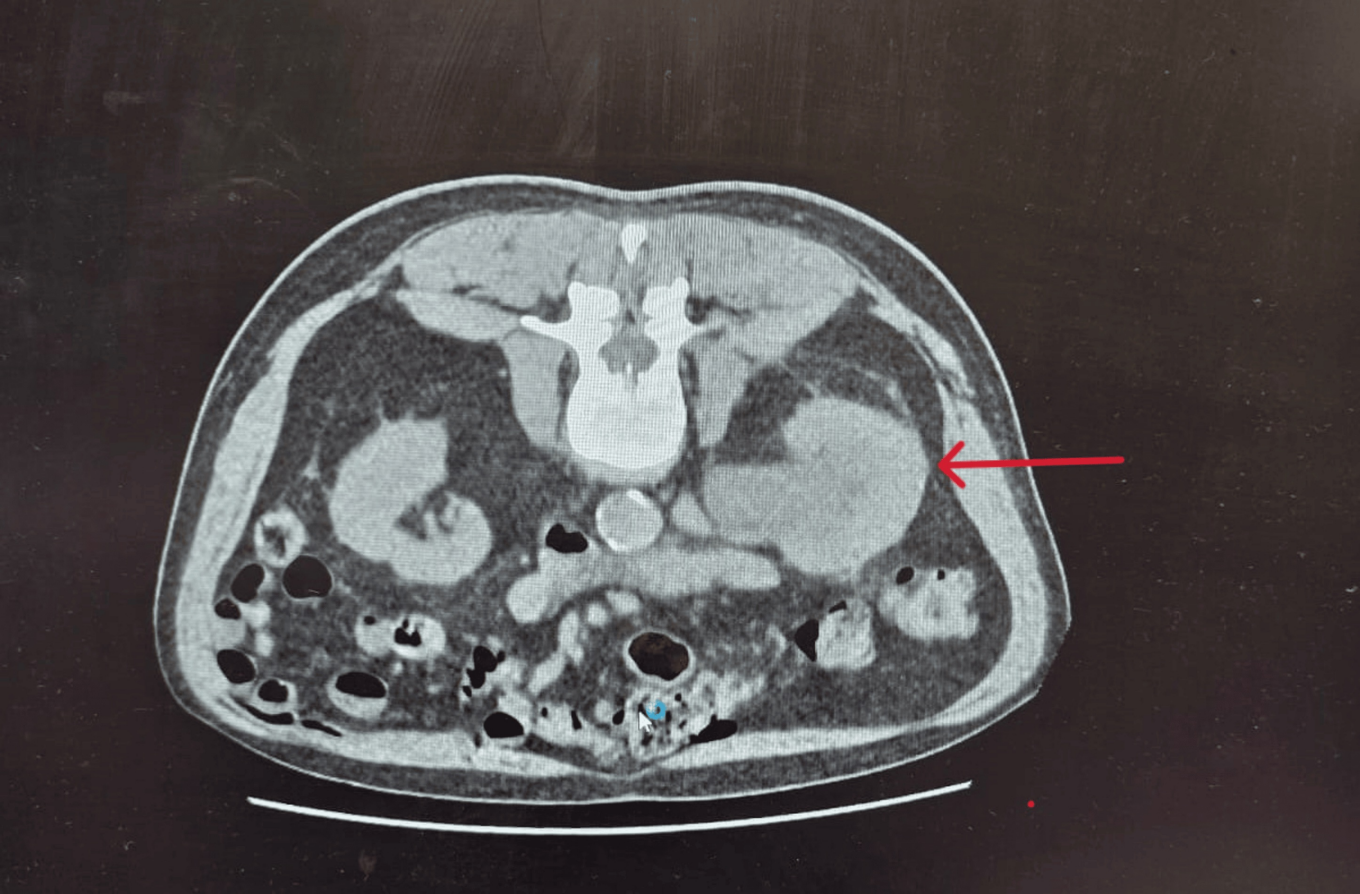
CT kidney-ureter-bladder (KUB) CT kidney-ureter-bladder (KUB) showing right hydronephrosis (red arrow) secondary to obstruction concerning for transitional cell carcinoma.

Clinical examination was unremarkable, and no palpable mass was felt. CT kidney-ureter-bladder (KUB) demonstrated a severe right-sided hydronephrosis with a transition point in the mid ureter concerning for transitional cell carcinoma (Figure 1).

Urinalysis showed microscopic haematuria (Table 2). Urine cytology revealed atypical squamous cells and keratin debris within a dense sea of neutrophils. Sections through a cell block confirmed the impression of malignant squamous cells, consistent with urothelial carcinoma with extensive squamous differentiation.

**Table 2:** Urinalysis report Urinalysis report showing microscopic haematuria.

Parameter	Result	Reference / Normal
Bilirubin	Negative	Negative
Urobilinogen	Normal	Normal
Ketones	Negative	Negative
Ascorbic Acid	Negative	Negative
Glucose	Normal	Negative/Normal
Protein	Negative	Negative
Erythrocytes (RBCs)	300 Ery/µL	0–25 Ery/µL
pH	Normal	4.5–8.0
Nitrite	Negative	Negative

**Figure 2:**
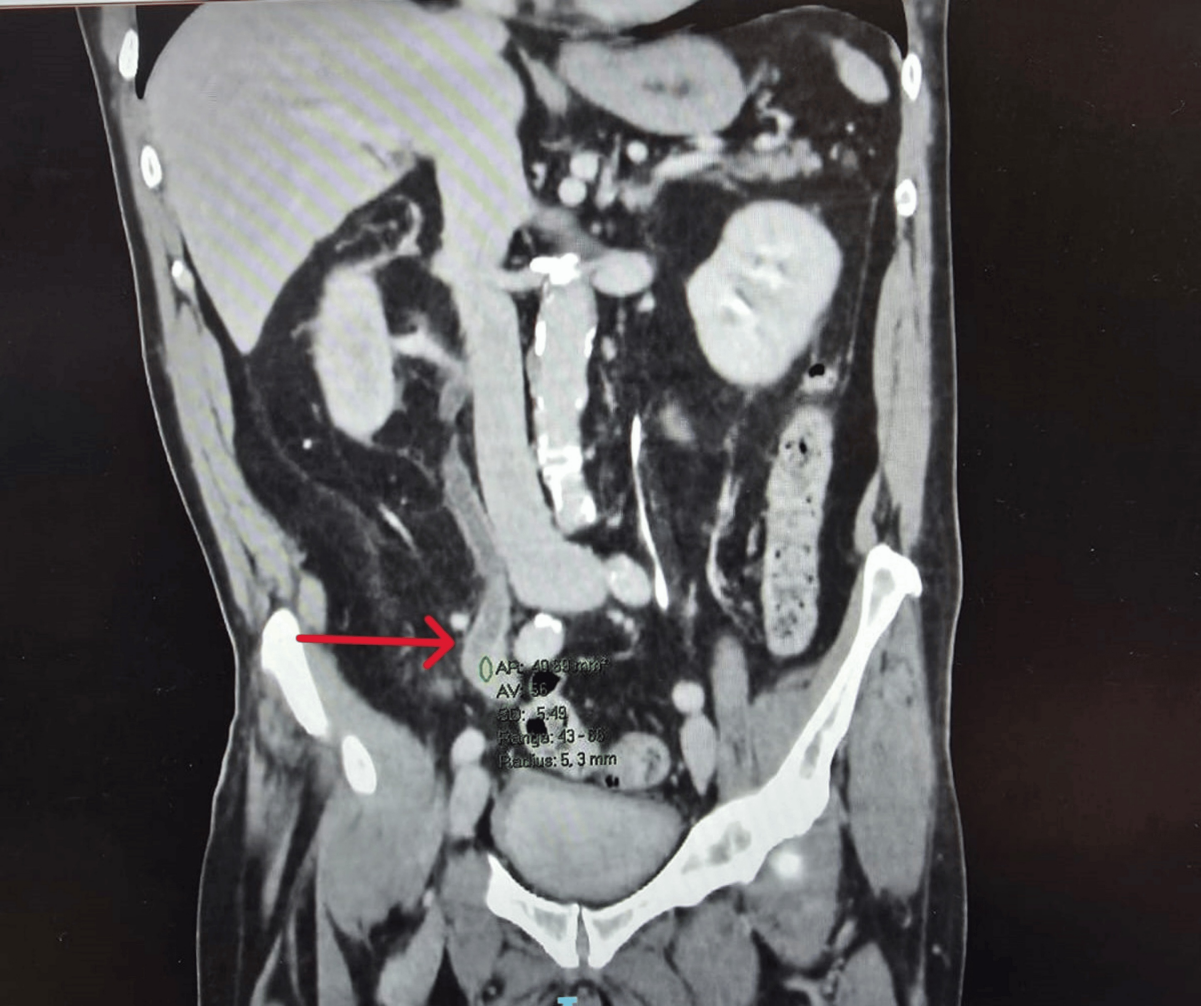
CT Urogram coronal view CT Urogram coronal view showing right hydroureter (Red Arrow) leading into right hydronephrosis.

**Figure 3:**
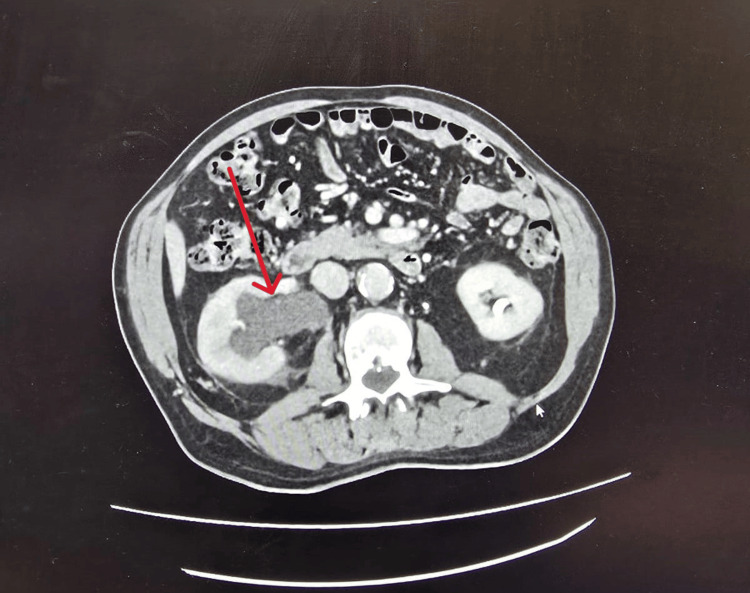
CT Urogram transverse section . CT Urogram transverse section showing right hydroureter (Red Arrow) leading into right hydronephrosis.

Subsequently, he underwent a CT urogram and CT thorax, which revealed a 21 mm mass lesion in the right ureter at L5 causing partial obstruction, leading to right hydronephrosis and hydroureter (Figures 2, 3). No evidence of distant metastasis was found. Radiological stage was T3 N0 M0 transitional cell carcinoma (TCC). A pre-operative cystoscopy was performed, which showed a trabeculated bladder but no bladder lesion.

The case was discussed at the local urology multidisciplinary team (MDT), and the patient underwent a robot-assisted laparoscopic right nephro-ureterectomy and pelvic lymph node dissection. The patient had an uneventful recovery and was discharged home the next day. 

Histopathology report confirmed moderately differentiated SCC, with a 0.65 mm clear margin at the distal ureter, with muscular invasion but no vascular or lymphatic invasion. The tumor size was 50 mm, and the tumor, node, metastasis (TNM) stage was pT3 N0 R0 V0. 

**Figure 4:**
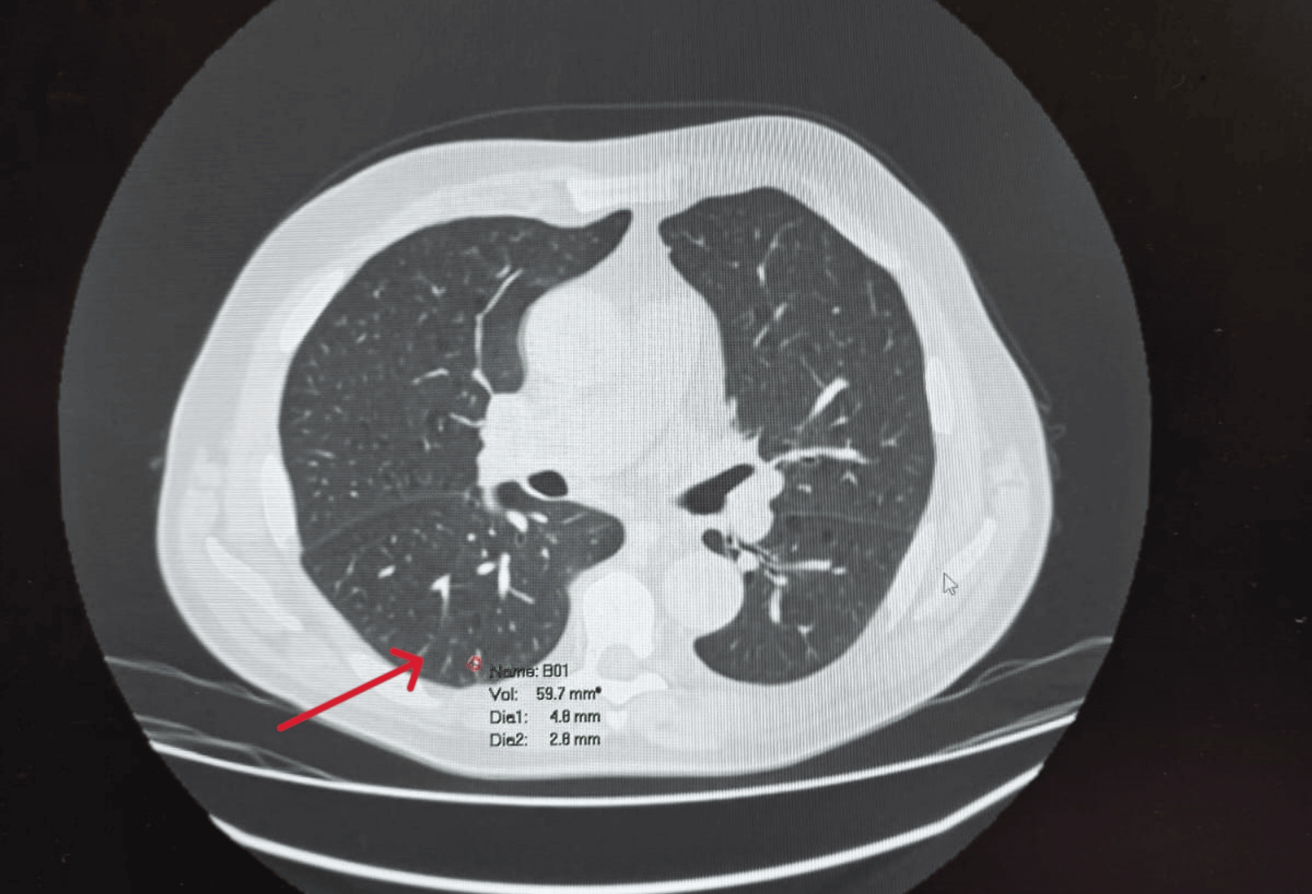
CT thorax CT thorax showing approximately 5mm lung nodule (Red Arrow) in the posterior aspect of right lower lobe.

Follow-up so far has revealed no recurrences, including at flexible cystoscopy three months postoperatively and surveillance CT. Surveillance CT thorax, however, revealed an indeterminate 5mm lung nodule, which is planned for surveillance (Figure 4).

## Discussion

This is a case report of a primary SCC in the urothelial tract with localized disease at presentation.

Primary upper tract SCC is extremely rare and only accounts for 0.5%-7 % of upper urinary tract carcinomas [7,8]. It usually presents in the sixth and seventh decades of life. It is a highly aggressive tumor that tends to metastasize to the bone, liver, and lungs. Because of the rapid progression of ureteral SCC, patients frequently present at an advanced stage, typically T3. The overall five-year survival rate is extremely low-less than 10%-and the median survival after diagnosis is short, averaging around five months.

Development of SCC is a gradual process that starts with pathological chronic inflammation, normally associated with chronic infection and urolithiasis. Other suggested causes include radiotherapy, vitamin A deficiency, and analgesic abuse. [9] This leads to staged histological changes of metaplasia, dysplasia, and eventually SCC.

Symptoms can include microscopic and macroscopic haematuria, loin pain, anaemia, weight loss, and a palpable abdominal mass. Mechanical obstruction can lead to hydronephrosis and stretching of the renal capsule, which causes pain [10]. Hypercalcemia, leukocytosis, and thrombocytopenia can also rarely present due to paraneoplastic syndromes [3]; however, these are less common.

Radiological features include hydronephrosis, a ureteric mass (extraluminal, intraluminal, or exophytic), and pelvic lymphadenopathy. Definitive diagnosis is with the histological presence of squamous differentiation and the presence of keratin pearls.

Our patient presented with flank pain and hydronephrosis detected on CT imaging, mimicking TCC. This underscores the limitations of imaging in differentiating SCC from other upper tract malignancies, as ureteric SCC may present radiologically with features identical to those of TCC. Cytology in our case revealed atypical squamous cells, which, although not definitive, supported suspicion for a non-urothelial malignancy

Given the limited number of cases of primary SCC, evidence for the best treatment options is limited. Radical nephro-ureterectomy and lymph node dissection are the main treatment modalities in localised disease. Neo-adjuvant and/or adjuvant chemotherapy and radiotherapy have also been used, but these treatment modalities have shown limited survival benefit [4].

In our case, robot-assisted nephro-ureterectomy achieved complete resection with negative margins, and the patient has remained recurrence-free at short-term follow-up, despite advanced local staging (pT3). This outcome challenges the traditional paradigm of mandatory adjuvant therapy in all patients with ureteric SCC and suggests that surgery alone may provide durable disease control in select cases with localized disease and negative surgical margins.

Our findings carry several implications. First, they underscore the diagnostic challenge posed by ureteric SCC, particularly in patients without conventional risk factors. Second, they highlight the pivotal role of CT urography in detecting subtle obstructive changes that may represent occult malignancy. Third, they demonstrate that minimally invasive robotic surgery can achieve favorable perioperative outcomes, including rapid recovery and clear oncological margins, even in elderly patients with multiple comorbidities. Finally, our case adds to the limited body of literature suggesting that carefully selected patients may be managed successfully with surgery alone, though longer follow-up is required to confirm sustained disease control.

## Conclusions

This case underscores that squamous cell carcinoma (SCC) of the ureter, despite being exceptionally rare, can arise even in the absence of traditional risk factors such as stones or chronic infection, necessitating heightened clinical suspicion during the evaluation of unexplained hydronephrosis. Definitive diagnosis relies on histopathological confirmation, with squamous differentiation and keratin pearl formation as hallmark features. Radical nephro-ureterectomy with lymphadenectomy remains the cornerstone of treatment in localized disease, while the role of adjuvant chemotherapy or radiotherapy remains uncertain, given limited evidence. Survival rates are generally poor due to late-stage presentation, yet our case demonstrates that complete surgical resection with negative margins may provide favorable short-term outcomes even without adjuvant therapy. Continued reporting of such cases is essential to strengthen the limited evidence base and guide future management strategies
